# Can ceftriaxone be omitted in the treatment of nongonococcal pelvic inflammatory disease?

**DOI:** 10.1177/09564624211004415

**Published:** 2021-04-28

**Authors:** James Ian McConville Ross, Polly Morton Roads, Jonathan D C Ross

**Affiliations:** 1 School of Medical Sciences, 5292 The University of Manchester, Manchester, UK; 2 Department of Sexual Health and HIV, 1732 Birmingham University Hospitals NHS Foundation Trust, Birmingham, UK

Dear Editor,

We read with interest the letter from Dabis regarding the decision by Watford Sexual Health to continue using doxycycline and metronidazole to treat non-gonococcal pelvic inflammatory disease (PID).^1^ As Dabis has noted, this is not consistent with the British Association of Sexual Health and HIV guideline, which recommends that a single dose of intramuscular ceftriaxone be given in addition to doxycycline/metronidazole.^2^ Dabis reports a retrospective study that suggests the omission of ceftriaxone did not affect patient outcomes. However, based on a number of factors, we would question whether this conclusion is valid.

Most importantly, clinical practice revolves around the principles of evidence-based medicine, where the best available evidence is used to guide clinical decision making. The report by Dabis is observational, retrospective, small (131 women) and has a high dropout rate between treatment and follow-up (48%), nor were the criteria for PID diagnosis or evaluation of cure standardised. The resultant findings are therefore at high risk of bias and, we would suggest, insufficient to justify a change in recommended practice. It remains possible that doxycycline/metronidazole alone may be as good as a cephalosporin-based regimen but, as outlined below, there are several reasons why this may not be the case.

Large randomised controlled trials have demonstrated that regimens which include a cephalosporin have high efficacy in PID.^2^ A similar level of evidence is not available for doxycycline/metronidazole without a cephalosporin. This is reflected not just in the UK PID guidelines but also in those from the USA, Europe and Australia,^3–5^ none of which recommends doxycycline plus metronidazole in isolation. The Cochrane review on the treatment of PID to which Dabis refers^6^ does not include a comparison of doxycycline/metronidazole regimens with and without ceftriaxone, and is therefore not helpful in addressing this issue.

In addition to the empirical evidence from clinical trials, the inclusion of a cephalosporin is also supported by a need to cover the relevant bacteria which cause PID. A wide variety of aerobic and anaerobic bacteria are associated with pelvic infection and ceftriaxone provides relevant broad spectrum activity, beyond just *Neisseria gonorrhoeae*.^7,8^ In contrast, the cover provided by doxycycline/metronidazole is limited.

Finally, Dabis suggests that two previous trials have found doxycycline/metronidazole to be not inferior when compared to other antibiotic regimens used in PID.^9,10^ However, this is of limited value since neither of the comparator regimens are a current standard of care and specifically do not include cephalosporin, limiting any conclusions about relative efficacy. A contemporaneous meta-analysis found the clinical and microbiological cure rate for doxycycline/metronidazole to be lower than alternative PID treatments.^11^

We agree that further high-quality trials are needed to guide the optimal choice of therapy for women with PID. However, the current best evidence, based on the microbiology of pelvic infection and large clinical trials, is that treatment with doxycycline/metronidazole should be combined with ceftriaxone.
